# Supplementary figures and images for: Free water: A marker of age-related modifications of the cingulum white matter and its association with cognitive decline
Source: PLoS One. 2020 Nov 20;15(11):e0242696. doi: 10.1371/journal.pone.0242696 (PMC7678997; doi:10.1371/journal.pone.0242696)

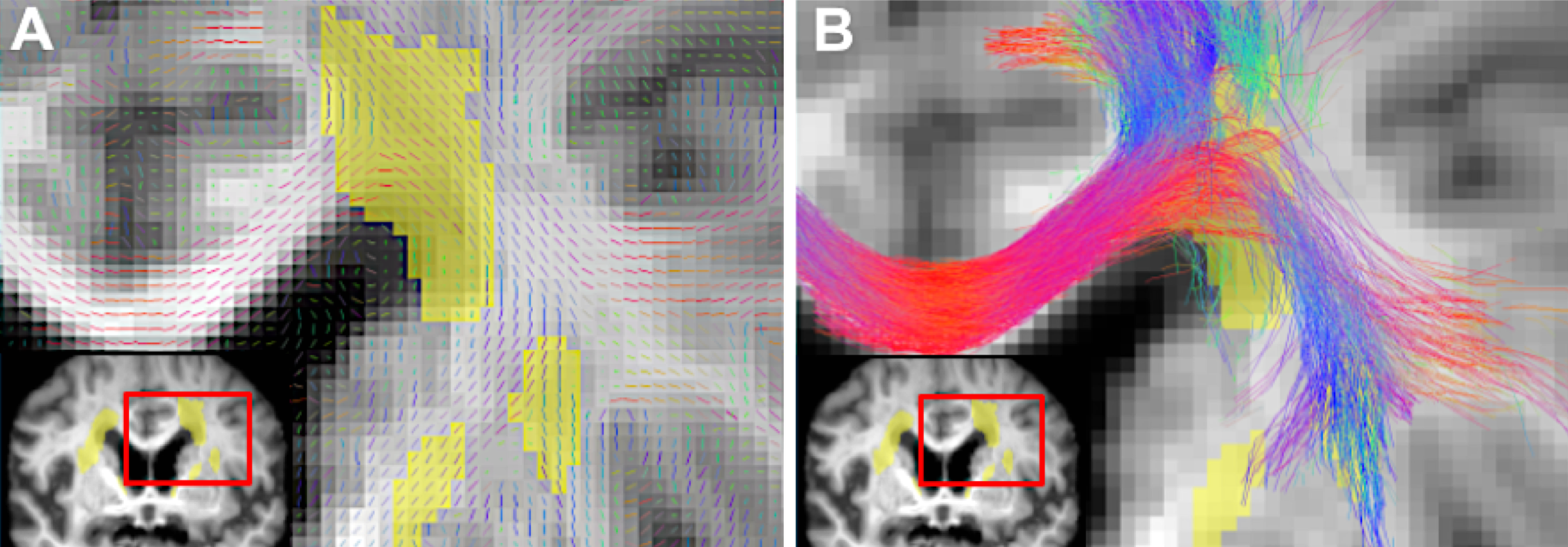

Supplement: S1 Fig — Yellow represents the white matter hyperintensity lesion mask displayed on the corresponding T1-weighted image. In A, the peak directions extracted from the fODF are preserved and coherent under the WMH. In B, the lesions do not impact the reconstruction of the Corpus Callosum. (TIF) [file pone.0242696.s001.tif]

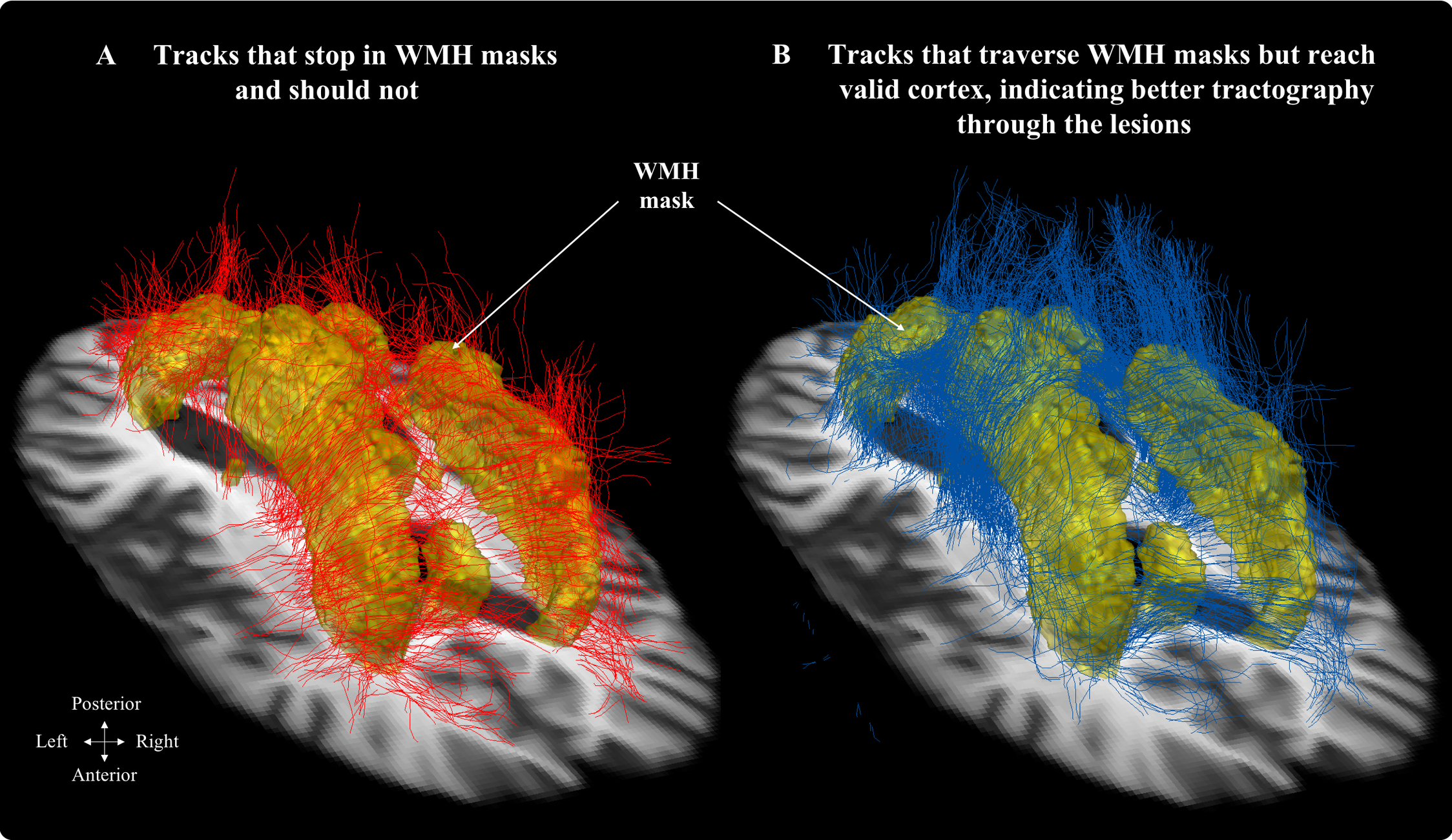

Supplement: S2 Fig — (A) In red, tracks that stop in WMH lesion instead of grey matter and should not. (B) In blue, tracks that cross the WMH mask and reach grey matter regions. WMH mask is represented in yellow, both displayed on the corresponding T1-weighted image. (TIF) [file pone.0242696.s002.tif]
