# Supplementary material for: Free water: A marker of age-related modifications of the cingulum white matter and its association with cognitive decline
Source: PLoS One. 2020 Nov 20;15(11):e0242696. doi: 10.1371/journal.pone.0242696 (PMC7678997; doi:10.1371/journal.pone.0242696)
Supplement: S1 Table — (DOCX) [file pone.0242696.s003.docx]

**S1 Table.** Relationship of diffusion measures with demographic, clinical, vascular and structural variables

|  | **FA** | **MD** | | **AD** | | **RD** | | **FAt** | | **MDt** | | **ADt** | | **RDt** | | **Free water** | |  |
| --- | --- | --- | --- | --- | --- | --- | --- | --- | --- | --- | --- | --- | --- | --- | --- | --- | --- | --- |
| Age ^b^ | 0.107 ^a^ | 0.014 | | 0.117 | | -0.001 | | 0.034 | | 0.061 | | 0.014 | | 0.001 | | -0.067 | |  |
|  | *0.504* | *0.466* | | *0.209* | | *0.849* | | *0.668* | | *0.512* | | *0.551* | | *0.838* | | *0.501* | |  |
| MMSE ^b^ | 0.175 | 0.196 | | 0.166 | | 0.006 | | 0.191 | | 0.201 | | 0.178 | | -0.053 | | -0.183 | |  |
|  | *0.832* | *0.200* | | *0.100* | | *0.158* | | *0.393* | | *0.102* | | *0.108* | | *0.838* | | *0.101* | |  |
| CES-D ^b^ | 0.136 | 0.031 | | 0.127 | | -0.076 | | 0.142 | | 0.077 | | 0.201 | | -0.058 | | -0.164 | |  |
|  | *0.099* | *0.458* | | *0.154* | | *0.402* | | *0.145* | | *0.379* | | *0.180* | | *0.226* | | *0.112* | |  |
| BMI ^b^ | -0.172 | -0.027 | | -0.103 | | 0.153 | | -0.208 | | -0.034 | | -0.212 | | -0.085 | | -0.213 | |  |
|  | *0.379* | *0.827* | | *0.649* | | *0.486* | | *0.099* | | *0.828* | | *0.346* | | *0.250* | | *0.096* | |  |
| Gender ^c^ | 521 | 460 | | 467 | | 471 | | 448 | | 521 | | 469 | | 462 | | 442 | |  |
|  | *0.807* | *0.213* | | *0.301* | | *0.472* | | *0.348* | | *0.733* | | *0.297* | | *0.527* | | *0.172* | |  |
| Education level ^c^ | 484 | 469 | | 516 | | 490 | | 492 | | 442 | | 530 | | 479 | | 520 | |  |
|  | *0.354* | *0.789* | | *0.816* | | *0.582* | | *0.823* | | *0.401* | | *0.712* | | *0.504* | | *0.509* | |  |
| Diabetes ^c^ | 49 | 63 | | 101 | | 42 | | 52 | | 67 | | 95 | | 39 | | 67 | |  |
|  | *0.125* | *0.442* | | *0.478* | | *0.113* | | *0.232* | | *0.369* | | *0.724* | | *0.157* | | *0.635* | |  |
| Hypertension ^c^ | 255 | 46 | | 260 | | 257 | | 262 | | 47 | | 259 | | 258 | | 49 | |  |
|  | *0.698* | *0.176* | | *0.346* | | *0.352* | | *0.691* | | *0.224* | | *0.356* | | *0.562* | | *0.232* | |  |
| BMI, Body Mass Index; CES-D, Center for Epidemiologic Studies-Depression scale; IST, Isaac Set Test; WMH, White Matter Hyperintensity  ^a^ Values are 𝜌 or U and *p-value* | | | | | | | | | | | | | | | | | | |
| ^b^ Spearman correlation, 𝜌 (rho) | | |  | |  | |  | |  | |  | |  | |  | |  |  |
| ^c^ Mann–Whitney U-tests, U | | |  | |  | |  | |  | |  | |  | |  | |  |  |
