# Supplementary material for: Free water: A marker of age-related modifications of the cingulum white matter and its association with cognitive decline
Source: PLoS One. 2020 Nov 20;15(11):e0242696. doi: 10.1371/journal.pone.0242696 (PMC7678997; doi:10.1371/journal.pone.0242696)
Supplement: S2 Table — (DOCX) [file pone.0242696.s004.docx]

**S2 Table.** Association between diffusion measures and WMH volumes

|  | **DTI** | | | | | | | **FW-corrected** | | | | | | | | | | |
| --- | --- | --- | --- | --- | --- | --- | --- | --- | --- | --- | --- | --- | --- | --- | --- | --- | --- | --- |
|  | **FA** | **MD** | | **AD** | | **RD** | | | **FAt** | **MDt** | **ADt** | | **RDt** | | **Free water** | |  |  |
|  | *𝜌* | *𝜌* | | *𝜌* | | *𝜌* | | | *𝜌* | *𝜌* | *𝜌* | | *𝜌* | | *𝜌* | |  |  |
| Total WMH volume (%) | -0.175 | 0.179 | | 0.001 | | **0.240*** | | | -0.194 | **0.212*** | -0.017 | | **0.257*** | | 0.046 | |  |  |
| WMH within Cingulum (%) | -0.147 | 0.168 | | 0.042 | | 0.180 | | | -0.213 | **0.231*** | 0.010 | | **0.265*** | | 0.105 | |  |  |
| WMH, White Matter Hyperintensity volumes were expressed as % of TIV | | | | | | | | | | | | | | | | | |  |
| Spearman’s correlation, *𝜌* | | | | |  | |  | | |  |  |  | |  | |  |  |  |
| * p < 0.05 FDR corrected | | |  | |  | |  | | |  |  |  | |  | |  |  |  |
