# Supplementary material for: Free water: A marker of age-related modifications of the cingulum white matter and its association with cognitive decline
Source: PLoS One. 2020 Nov 20;15(11):e0242696. doi: 10.1371/journal.pone.0242696 (PMC7678997; doi:10.1371/journal.pone.0242696)
Supplement: S3 Table — (DOCX) [file pone.0242696.s005.docx]

**S3 Table.** Relationship between cingulum diffusion measures and verbal fluency score (IST) before (conventional DTI) and after FW-correction in model adjusted for total WMH volume.

| DTI | | | | | | | |
| --- | --- | --- | --- | --- | --- | --- | --- |
|  |  | IST 15s | | IST 30s | | IST 60s | |
|  |  | *β* | *R^2^* | *β* | *R^2^* | *β* | *R^2^* |
| Model adjusted for total WMH | FA | 0.022 | 0.023 | 0.040 | 0.052 | 0.069 | 0.067 |
|  | MD | -0.267 | 0.020 | -0.218 | 0.010 | -0.147 | 0.09 |
|  | AD | -0.172 | 0.054 | -0.109 | 0.062 | -0.029 | 0.062 |
|  | RD | -0.119 | 0.036 | -0.110 | 0.062 | -0.121 | 0.075 |
|  |  |  |  |  |  |  |  |
| **FW-corrected** | | | | | | | |
|  |  | IST 15s | | IST 30s | | IST 60s | |
|  |  | *β* | *R^2^* | *β* | *R^2^* | *β* | *R^2^* |
| Model adjusted for total WMH | FAt | 0.035 | 0.044 | 0.072 | 0.061 | 0.071 | 0.07 |
|  | MDt | **-0.401*** | **0.20** | **-0.341*** | **0.17** | -0.189 | 0.11 |
|  | ADt | -0.067 | 0.10 | -0.185 | 0.10 | -0.097 | 0.08 |
|  | RDt | -0.224 | 0.11 | -0.213 | 0.11 | -0.173 | 0.09 |
|  | FW | **-0.333*** | **0.15** | **-0.275*** | **0.14** | -0.213 | 0.10 |

*β*, standardized coefficient regression adjusted for age, cingulum white matter volume and total WMH volume

R^2^, R square value

* p < 0.05 FDR corrected
