# Supplementary material for: Free water: A marker of age-related modifications of the cingulum white matter and its association with cognitive decline
Source: PLoS One. 2020 Nov 20;15(11):e0242696. doi: 10.1371/journal.pone.0242696 (PMC7678997; doi:10.1371/journal.pone.0242696)
Supplement: S4 Table — (DOCX) [file pone.0242696.s006.docx]

**S4 Table.** Relationship between cingulum diffusion measures and verbal fluency score (IST) before (conventional DTI) and after FW-correction in model unadjusted and adjusted for cingulum WMH volume.

| DTI | | | | | | | |
| --- | --- | --- | --- | --- | --- | --- | --- |
|  |  | IST 15s | | IST 30s | | IST 60s | |
|  |  | *β* | *R^2^* | *β* | *R^2^* | *β* | *R^2^* |
| Model unadjusted for cingulum WMH^b^ | FA | 0.003 | 0.001 | 0.04 | 0.002 | 0.061 | 0.004 |
|  | MD | **-0.219*** | **0.091** | -0.24 | 0.06 | -0.178 | 0.03 |
|  | AD | -0.203 | 0.089 | -0.18 | 0.03 | -0.096 | 0.009 |
|  | RD | -0.146 | 0.021 | -0.147 | 0.02 | -0.144 | 0.02 |
| Model adjusted for cingulum WMH^b^ | FA | 0.015 | 0.03 | 0.042 | 0.05 | 0.063 | 0.033 |
|  | MD | -0.211 | 0.1 | -0.211 | 0.09 | -0.141 | 0.09 |
|  | AD | -0.221 | 0.092 | -0.150 | 0.08 | -0.058 | 0.08 |
|  | RD | -0.151 | 0.06 | -0.136 | 0.07 | -0.131 | 0.08 |
|  |  |  |  |  |  |  |  |
| **FW-corrected** | | | | | | | |
|  |  | IST 15s | | IST 30s | | IST 60s | |
|  |  | *β* | *R^2^* | *β* | *R^2^* | *β* | *R^2^* |
| Model unadjusted for cingulum WMH^a^ | FAt | -0.055 | 0.003 | 0.037 | 0.001 | 0.049 | 0.002 |
|  | MDt | **-0.368*** | **0.14** | **-0.308*** | **0.1** | -0.226 | 0.06 |
|  | ADt | -0.116 | 0.013 | -0.211 | 0.05 | -0.125 | 0.016 |
|  | RDt | **-0.333*** | **0.12** | -0.162 | 0.03 | -0.148 | 0.022 |
|  | FW | **-0.353*** | **0.13** | **-0.241*** | **0.1** | -0.126 | 0.2 |
| Model adjusted for cingulum WMH^b^ | FAt | -0.064 | 0.04 | 0.013 | 0.06 | 0.023 | 0.07 |
|  | MDt | **-0.365*** | **0.17** | **-0.280*** | **0.13** | -0.187 | 0.1 |
|  | ADt | -0.110 | 0.05 | -0.198 | 0.09 | -0.108 | 0.09 |
|  | RDt | **-0.321*** | **0.14** | -0.148 | 0.07 | -0.116 | 0.09 |
|  | FW | **-0.333*** | **0.16** | **-0.226*** | **0.12** | -0.100 | 0.085 |

*^a^ β*, standardized coefficient regression adjusted for age and cingulum white matter volume

*^b^ β*, standardized coefficient regression adjusted for age, cingulum white matter volume and cingulum WMH volumes

R^2^, R square value

* p < 0.05 FDR corrected
