# Supplementary material for: Free water: A marker of age-related modifications of the cingulum white matter and its association with cognitive decline
Source: PLoS One. 2020 Nov 20;15(11):e0242696. doi: 10.1371/journal.pone.0242696 (PMC7678997; doi:10.1371/journal.pone.0242696)
Supplement: S1 Appendix — (DOCX) [file pone.0242696.s007.docx]

**Free water: a marker of age-related modifications of the cingulum white matter and its association with cognitive decline**

*Manon Edde^1,2¶*^, Guillaume Theaud^6¶^, François Rheault^6^, Bixente Dilharreguy^2^, Catherine Helmer^4^, Jean-François Dartigues ^4,5^, Hélène Amieva^4^, Michèle Allard^1,2,5^, Maxime Descoteaux^6^****^&^****, Gwénaëlle Catheline^1,2,3^****^&^***

**1. Correction of tractography with white matter hyperintensities (WMH)**

In elderly individuals, the presence of WMH can lead to a misclassification during the segmentation process considering that a voxel within WMH could be attributed to grey matter. This misclassification is an issue in tractography since tracking and seeding masks are generated from gray and white matter maps. Therefore, this misclassification can lead to a premature ending of tracks in WMH instead of grey matter. Another important issue of WMH lesions in tractography is the decrease of FA inside WM lesions that could also lead to premature ending of tracks. However, we observed that the principal directions under the lesion mask seem to be preserved and remain coherent and that tractography should be allowed to explore these regions (S1 Figure). To determine the effect of WMH on tractography, we produced two whole brain tractograms: a first one without WMH correction and a second one with WMH correction.

The seeding and tracking masks were modified to include the WMH mask, as previously described [1]. Briefly, as described in the Methods section, WMH maps were segmented using the Lesion Segmentation Tool (LST, v2.0; [2], visually inspected and registered to the corresponding dMRI images using ANTs registration tool. Finally, WMH maps were added in the inclusion and exclusion masks for each subject. We observed that without correction 24.4% of tracks stopped in WMH mask (S2A Figure), while with correction the percentage of tracks crossing the WMH mask was improved (13.4% *versus* 29.2%, without and with correction respectively, p < 0.05, S2B Figure). According to this observation, the cingulum bundle was reconstructed with a tractography pipeline that includes a WMH mask to limit the negative impact of hyperintensities on fiber tracking algorithms.

**2. Association between diffusion measures and clinical or vascular variables**

None of the cingulum diffusion measures were associated with demographic, clinical and vascular variables (p > 0.05, S1 Table).

**3. Association between diffusion measures and WMH volumes**

We observed a significant correlation between cingulum diffusion measures and total WMH volume (p<0.05; S2 Table). More specifically, higher WMH volumes were associated with a higher RD (p-FDR = 0.043) using conventional DTI measures. No association was observed for cingulum WMH.

After FW-correction, higher total WMH and cingulum WMH volumes were related to higher MDt (p-FDR = 0.04 and 0.038, for total and cingulum WMH volumes respectively) and RDt (p-FDR = 0.033 and 0.029, respectively) along the cingulum bundle. No association was observed between free water content and WMH volumes in our subjects.

**4. Impact of WMH volumes on the associations with cognition**

*4.1. Impact of total WMH volume on the association between cingulum diffusion measures and changes in verbal fluency.*

In regression models adjusted for total WMH burden, no association were found with conventional DTI measures. After FW-correction, MDt and free water content correlations remained significant while the association with RDt did not reach significance (p = 0.067).

*4.2. Impact of WMH within the cingulum bundle on the association between cingulum diffusion measures and changes in verbal fluency.*

Interestingly, despite the fact that only 3% of the cingulum crossed areas with WMH lesions in our population, this small WMH burden had an impact on the association between cingulum diffusion measures and changes in verbal fluency in a model adjusted for age and white matter volume of the cingulum (p < 0.05 FDR-corrected, S4 Table). More specifically, for conventional DTI measures, lower MD was associated with IST decline at 15 seconds. No association was observed with FA, RD and AD. After FW-correction, higher MDt and RDt were associated with IST decline at 15 and 30 seconds. No association was observed with FAt and ADt. High free water content was associated with changes in IST score at 15 and 30 seconds.

**Supplementary References**

1. Theaud G, Dilharreguy B, Catheline G, Descoteaux M. Impact of white matter hyperintensities on tractography. In 2016.

2. Schmidt P, Gaser C, Arsic M, Buck D, Förschler A, Berthele A, et al. An automated tool for detection of FLAIR-hyperintense white-matter lesions in Multiple Sclerosis. NeuroImage. 15 févr 2012;59(4):3774‑83.
